# Genomic alterations in two patients with esophageal carcinosarcoma identified by whole genome sequencing: a case report

**DOI:** 10.1186/s40792-024-01978-8

**Published:** 2024-08-19

**Authors:** Masazumi Inoue, Yasuhiro Tsubosa, Sumiko Ohnami, Kazunori Tokizawa, Shuhei Mayanagi, Keiichi Ohshima, Kenichi Urakami, Shumpei Ohnami, Takeshi Nagashima, Ken Yamaguchi

**Affiliations:** 1 Division of Esophageal Surgery, Shizuoka Cancer Center Hospital, 1007 Shimonagakubo, Nagaizumi-Cho, Sunto-Gun, Shizuoka 411-8777 Japan; 2 Cancer Diagnostics Research Division, Shizuoka Cancer Center Research Institute, 1007 Shimonagakubo, Nagaizumi-Cho, Sunto-Gun, Shizuoka 411-8777 Japan; 3 Medical Genetics Division, Shizuoka Cancer Center Research Institute, 1007 Shimonagakubo, Nagaizumi-Cho, Sunto-Gun, Shizuoka 411-8777 Japan; 4 SRL, Inc, Akasaka Intercity Air, 1-8-1 Akasaka, Minato-Ku, Tokyo 107-0052 Japan; 5 Shizuoka Cancer Center, 1007 Shimonagakubo, Nagaizumi-Cho, Sunto-Gun, Shizuoka 411-8777 Japan

**Keywords:** Esophageal carcinosarcoma, Whole genome sequencing, *FANC* germline variants

## Abstract

**Background:**

Esophageal carcinosarcoma (ECS) is a relatively rare malignancy, accounting for < 1% of all esophageal cancers. Its etiopathogenesis remains unknown. This study analyzed the genomic abnormalities in sarcomatous tumors from two patients undergoing subtotal esophagectomy using whole genome sequencing to elucidate the key characteristics of ECS.

**Case presentation:**

We identified *TP53* driver mutations, copy number gains in 11q13 (including *CCND1*), and Apolipoprotein B mRNA editing enzyme catalytic polypeptide (APOBEC) signature enrichment in both ECS patients. Along with common genetic abnormalities, we identified *CDKN2A* driver mutations in case 1 and *RAC1*, *NOTCH1,* and *TTC28* as novel fusion gene partners of *MECOM* in case 2. Notably, we detected germline pathogenic variant in Fanconi anemia (FA) complementation group I (*FANCI*) and group G (*FANCG*), which are involved in repairing DNA double-strand breaks by homologous recombination, for the first time, in ECS blood samples. These germline variants were truncating-type, Lys1221fs of *FANCI* (rs1567179036) for case 1 and Gln365Ter of *FANCG* (rs121434426) for case 2. We also identified somatic changes in cancer-associated pathways, such as *PI3K/Akt/mTOR*, cell cycle, and *NOTCH* signaling pathways, and structural chromosomal defects such as chromosome doubling.

**Conclusions:**

Our findings indicate that therapeutic drugs targeting the activation signal or FA pathway might be effective in treating ECS, however, their therapeutic significance should be elucidated in future studies.

**Supplementary Information:**

The online version contains supplementary material available at 10.1186/s40792-024-01978-8.

## Background

Esophageal carcinosarcoma (ECS) is a relatively rare cancer, accounting for < 1% of all esophageal malignancies [1, 2]. Histopathological findings of sarcomatoid cells are presumably altered forms of esophageal squamous cell carcinoma (ESCC). Due to the low incidence of ECS, no treatment guidelines have been established yet. Although the histopathological features are well established, the genomic data on ECS is lacking. Here, we pioneered reporting the whole-genome sequencing (WGS) for the genomic analysis of ECS.

## Case presentations

### Case 1

**Fig. 1:**
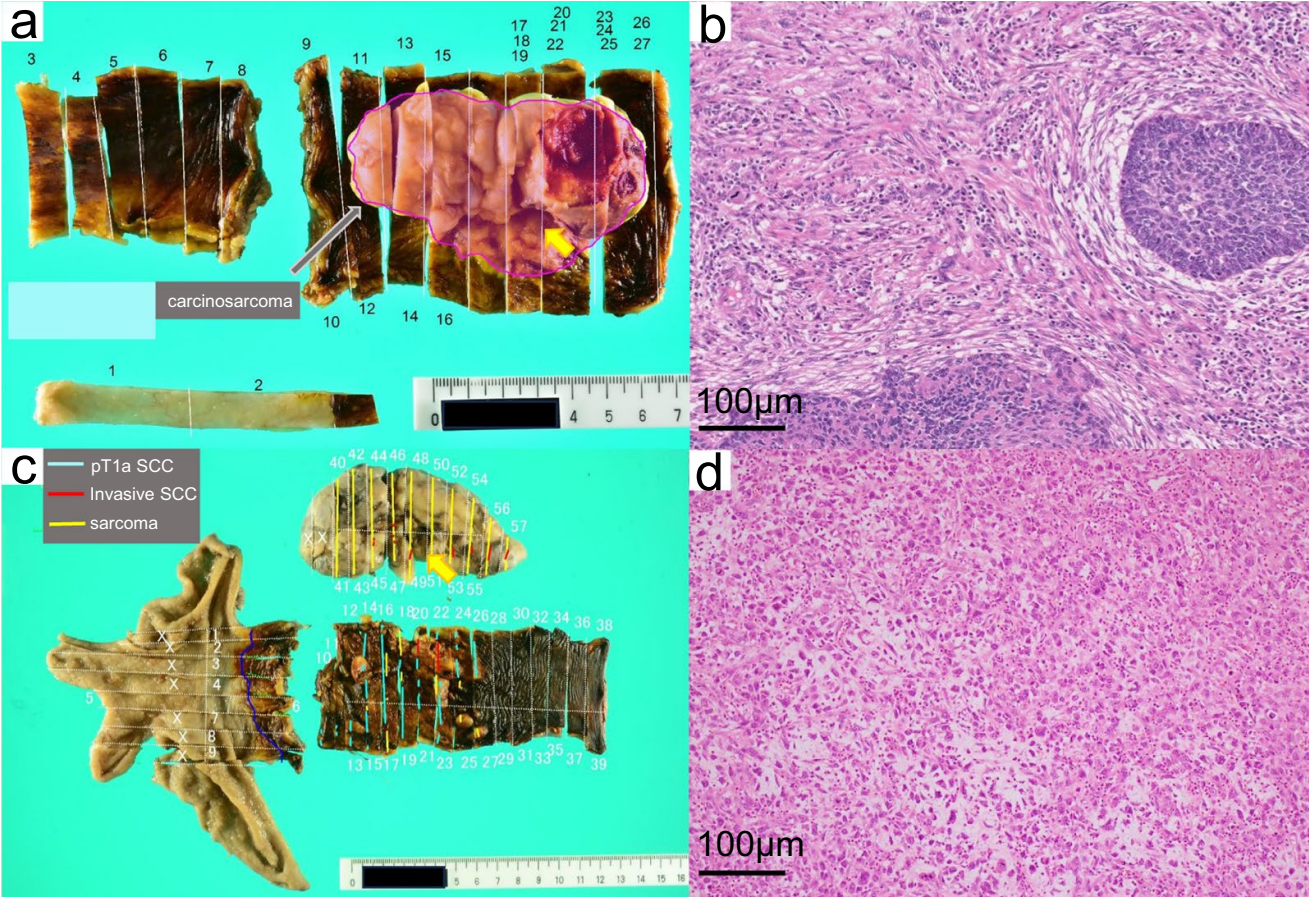
Resected specimen and pathological findings of the resected specimen close to the region analyzed by WGS. **a** Resected specimen with mapping of the location of carcinosarcoma in case 1. The yellow arrow shows the region analyzed by WGS. **b** Resected specimen close to the region analyzed by WGS in case 1 shows the spindle-shaped sarcoma-like cells around the stroma of the cancer. **c** Resected specimen with mapping of the location of sarcoma, invasive SCC and pT1a SCC in case 2. The yellow arrow shows the region analyzed by WGS. **d** Resected specimen close to the region analyzed by WGS in case 2 shows mucosarcoma-like and chondrosarcoma-like differentiation

A 79-year-old man presenting with dysphagia was diagnosed with ESCC using upper gastrointestinal endoscopy and referred to our hospital. Upper gastrointestinal endoscopic examination confirmed an irregular elevated tumor from the upper to the middle thoracic esophagus. Pathological examination of the biopsy specimens revealed a poorly differentiated carcinoma. Poorly differentiated squamous cell carcinoma and carcinosarcoma were the differential diagnoses. Computed tomography (CT) revealed a tumor filling the esophageal lumen from the upper to the middle thoracic esophagus, with no invasion into the surrounding organs. CT also revealed swelling of the subcarinal and main bronchial lymph nodes. The case was clinically diagnosed as ESCC cT3N1M0 cStage III based on the Union for International Cancer Control TNM classification scheme (UICC TNM 8th edition) [3]. No preoperative treatment was administered, and the patient underwent subtotal esophagectomy with three-field lymph node dissection. Pathological examination of the resected specimen revealed basaloid squamous cell carcinoma with sarcomatous change, and the final pathological diagnosis was ECS pT3N0M0 pStage IIB (UICC TNM 8th edition). We used the sarcoma portion of the resected specimen for subsequent genomic analysis (Fig. 1a, b).

### Case 2

A 73-year-old man presenting with dysphagia and pain was diagnosed with esophageal cancer using upper gastrointestinal endoscopy and referred to our hospital. Upper gastrointestinal endoscopy revealed an irregular elevated tumor from the middle to the lower thoracic esophagus. Pathological examination of the biopsy specimens revealed ECS. CT revealed a pedunculated tumor filling the esophageal lumen from the middle to the lower thoracic esophagus, with no invasion into the surrounding organs. There were no swollen lymph nodes. The case was clinically diagnosed as ECS cT2N0M0 cStage II (UICC TNM 8th edition). Due to her poor respiratory function, no preoperative treatment was administered, and a transhiatal subtotal esophagectomy with minimal abdominal lymph node dissection was performed. Pathological examination of the resected specimen revealed ECS, and the final pathological diagnosis was ECS pT1bN2M0 pStage IIIA (UICC TNM 8th edition). Subsequently, we performed genomic analysis on the sarcoma portion of the resected specimen (Fig. 1c, d).

### Ethical statement

All experimental protocols were approved by the Institutional Review Board of the Shizuoka Cancer Center (Authorization numbers 25–33). Written informed consent was obtained from all patients before they participated in this study. All experiments using clinical samples were performed following the approved Japanese ethical guidelines (human genome/gene analysis research, 2017, provided by the Ministry of Health, Labor, and Welfare (https://www.mhlw.go.jp/stf/seisakunitsuite/bunya/holabunya/kenkyujigyou/i-kenkyu/index.html).

### Genomic analysis

We performed WGS using the blood samples taken from an arterial line during surgery and fresh surgical specimens collected from resected specimen after surgery. Simultaneously, we performed gene expression profiling (GEP) using matched tumors and adjacent normal tissues from the patients. Regarding the tumor samples, a pathologist confirmed that the tumor content was 50% or more.

WGS analysis was conducted using the Illumina NovaSeq 6000 sequencing system (Illumina Inc., San Diego, CA, USA). Briefly, 1 µg of DNA was used for library preparation with the TruSeq DNA Polymerase Chain Reaction Free Sample Preparation Kit (Illumina). Paired tumor and blood libraries were pooled per lane following the manufacturer’s instructions. Genetic variants were identified using the DRAGEN small variant caller, annotated with gene consequence using an Ensembl variant effect predictor. The actionability of the variants was evaluated using an in-house annotation pipeline similar to that used in whole exome sequencing (WES) analysis [4]. Moreover, we used the “Rtsne” package (https://github.com/jkrijthe/Rtsne) for t-distributed stochastic neighbor embedding (t-SNE) analysis of the GEP data set [5].

### Results of genomic analysis

**Fig. 2:**
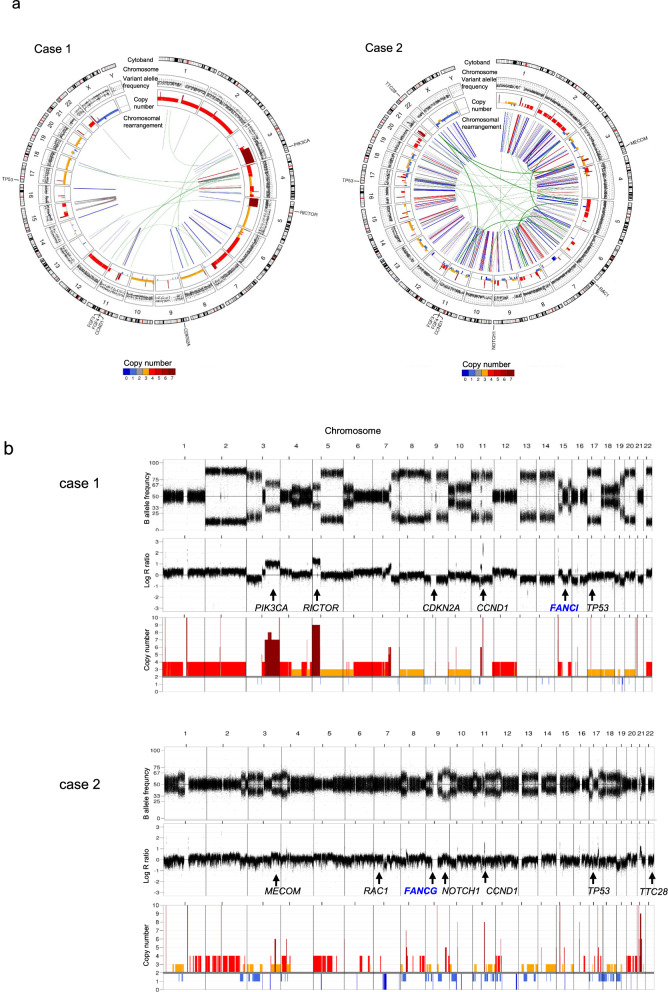

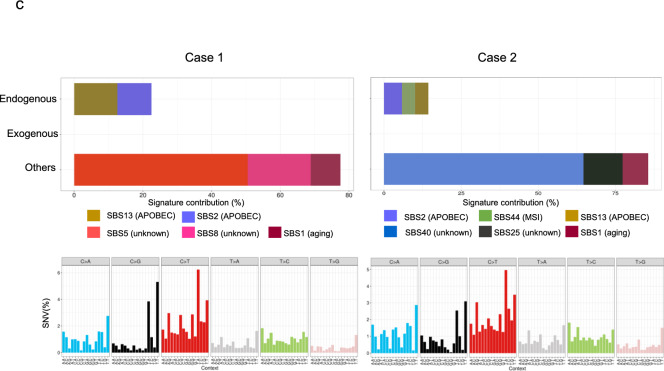
WGS of an ECS sample with structural aberrations. **a** Circos plot shows the structural variations in patients with ECS. The inner ring indicates the copy number variations (red: gain; blue: loss). **b** B allele frequency (top), log R ratio (middle), and copy number variation (bottom) in each chromosome. Arrows indicate the copy number variations in gain and loss peaks with putative cancer driver genes identified in patients with ECS. **c** Mutation signature and distribution of somatic single-nucleotide variants (SNVs) in patients with ECS identified by WGS (bottom)

As shown in Fig. 2, WGS analysis revealed genomic changes in both ECS patients. We identified structural chromosomal aberrations resulting in copy number (CN) gains in 3q26 (including *PIK3CA*), 5p13 (including *RICTOR*), and 11q13 (including *CCND1*), in case 1. In case 2, a potential fusion gene, *TTC28-MECOM*, was detected as a novel partner of *MECOM*, along with the *CCND1* CN gains (Supplementary Fig. 1) observed in case 2. The *TTC28-MECOM* fusion gene with CN gains of *MECOM* in tumor specimen was validated by PCR using the primer set from *TTC28* and *MECOM* (Supplementary Fig. 1). The median tumor mutational burden (TMB) in case 1 and case 2 were 4.33 mutations/Mb and 4.82 mutations/Mb, respectively.

Somatic mutation data included 78 nonsynonymous mutations (63 missense, 4 nonsense, and 7 frameshift insertions/deletions, and 4 inframe insertions/deletions) in case 1 and 72 nonsynonymous mutations (57 missense, 7 nonsense, and 6 frameshift mutations, and 2 insertions/deletions) in case 2 identified using WGS. All identified genes are listed in Supplementary Table 1.

Genomic abnormalities in well-annotated cancer driver genes were commonly identified in *TP53* mutations and *CCND1* CN gain (Table 1). Both cases were characterized by mutational signatures 2 and 13 associated with the *APOBEC* family, similar to our previous ESCC study [5].

**Table 1:** Detection of driver gene mutations and copy number alterations by whole-genome sequencing

Gene	Germline/Somatic	Genomic alterations	ClinVar^a^	Protein change	Function^b^
Patient 1					
*FANCI*	Germline	Flameshift	Pathogenic	Lys1221fs	Genome maintenance
*CDKN2A*	Somatic	Flameshift		Leu16fs	Cell cycle
*TP53*	Somatic	Flameshift		Arg335fs	Genome maintenance
*CCND1*	Somatic	CN gain			Cell cycle
*PIK3CA*	Somatic	CN gain			Cell growth
*RICTOR*	Somatic	CN gain			Cell growth
Patient 2					
*FANCG*	Germline	Nonsense	Pathogenic	Gln356Ter	Genome maintenance
*RAC1*	Somatic	Missense		Pro29Ser	Cell growth
*NOTCH1*	Somatic	Nonsense		Gln885Ter	Differentiation
* TP53*	Somatic	Missense		Tyr126Asp	Genome maintenance
* CCND1*	Somatic	CN gain			Cell cycle
* TTC28-MECOM*	Somatic	Fusion			

^a^ClinVar: *Nucleic Acids Res., 2016;44:D862-D868*

^b^Function: *Cancer Science 2019, *https://doi.org/10.1111/cas.14290

**Fig.3:**
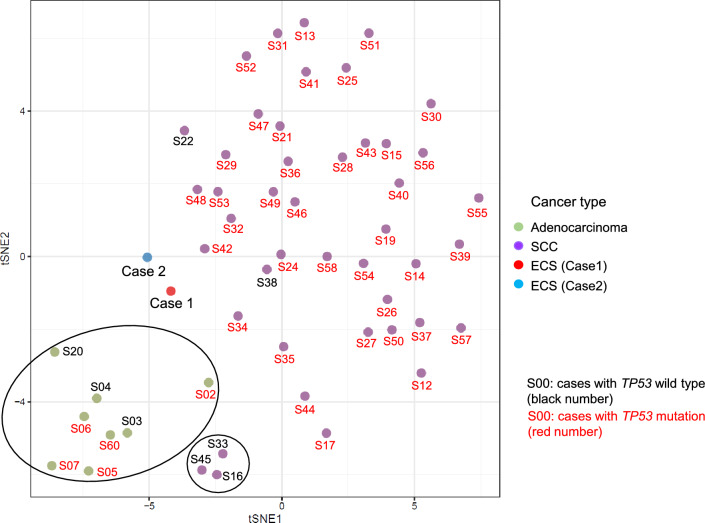
t-Distributed stochastic neighbor embedding analysis of the total gene expression data of ECS. Red circles indicate the ECS samples in this study. Green and purple circles, respectively, indicate the gene expression profiling data of adenocarcinoma and squamous cell carcinoma slightly modified from a previous report [5]. Black and red numbers indicate patients harboring wild-type and mutated *TP53*, respectively

Figure 3 shows the two-dimensional t-SNE analysis results of GEP data in ECS for 29,833 genes registered on the Entrez Gene Database using the “Rtsne” package. ESCC and esophageal adenocarcinoma (EAC) information were obtained from the GEP data based on our previous study [5]. We found that the gene expression patterns in ECS differed from those in ESCC or EAC. Notably, ECS cases exhibited expression patterns analogous to EAC and ESCC cases without *TP53* mutations, although this study identified a *TP53* mutation.

**Fig.4:**
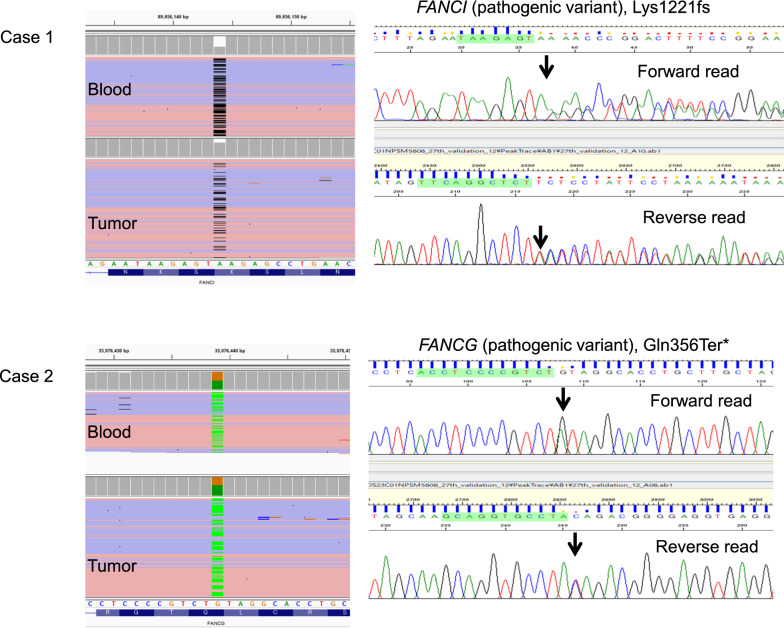
Visualization of variants using the integrative genomics viewer (left) and their validation using Sanger sequencing (right). *FANCI* pathogenic frameshift variant (Lys1221fs) and *FANCG* pathogenic nonsense variant (Gln356Ter*). Arrows indicate the variants

Germline analysis of blood samples revealed pathogenic truncated-type variants in the *FANC* group, including *FANCI* Lys1221fs (rs1567179036) and *FANCG* Gln356Ter (rs21434426) in cases 1 and 2, respectively. A previous study reported *FANCI* and *FANCG* variant minor allele frequency as 0.00003 and 0.00037, respectively, in a Japanese population [6]. These variants were visually inspected using an integrative genomics viewer and validated by Sanger sequencing (Fig. 4).

Our results indicated that pathogenic germline variants of Fanconi anemia (FA) complementation group genes are potentially involved in the ECS carcinogenesis.

## Discussion

This study reports the first case of genomic analysis of ECS using WGS. We detected amplifications of *PIK3CA*, *RICTOR*, and *CCND1* in somatic cells, activating the PI3K/Akt/mTOR and cell cycle signaling pathways [7]. This subsequently promoted cell growth, angiogenesis, and differentiation, thereby facilitating tumor progression. Furthermore, chromothripsis-related structural aberrations may be involved in the pathogenesis of ECS and associated with gains in chromosomes 3q, 5p, and 11q, and loss of chromosome 19q. Along with some structural chromosomal defects, our WGS analysis revealed *TP53, CDKN2A, RAC1,* and *NOTCH1* driver mutations and *KMT2D, FAT1/2,* and *EP400* mutations in the ECS samples, which is partially consistent with previous WES results [7, 8]. Moreover, we identified a novel *TTC28-MECOM* fusion gene in the ECS patient with CN gains of *MECOM* (Supplementary Fig. 2). *MECOM* is a transcriptional regulator implicated in leukemogenesis that can be rearranged with various partner genes, such as *H2AFY*, *RUNX1*, and *GATA2* [9]. *TTC28-MECOM* fusion may modulate the carcinosarcoma phenotype in ECS carcinogenesis; however, this requires functional verification in future studies.

ECS histologically consists of neoplastic squamous and sarcomatous spindle cells, with the sarcomatoid component predominating in most cases [10]. The prognosis of ECS is reportedly better than that of esophageal squamous cell carcinoma [11, 12]. Another study revealed that the incidence was nearly equal to that of conventional esophageal carcinoma [8]. Regarding the genetic characteristics of ECS, it has been reported that *TP53* mutations are frequent, similar to ESCC, however, there have been few analyses using next-generation sequencing. Tsuyama et al. identified *TP53* (6/6) and *PTEN* (2/6) mutations in six ECS cases using WES, and detected LOH at the *INI1* locus [8]. However, our analysis revealed a more detailed genetic signature for ECS by simultaneously analyzing WGS and GEP in this study, which may enhance our understanding of potential therapeutic targets for ECS.

Notably, for the first time, truncated-type pathogenic mutations in *FANCI* and *FANCG* were detected and confirmed by Sanger sequencing in ECS blood samples. *FANC* genes are important in repairing double-strand breaks by homologous recombination. FA repair pathway is important in maintaining genome stability [13]. These findings indicate that inactivation events are involved in the FA pathway, resulting in chromothripsis [14].

Dysfunctional FA gene products are associated with DNA damage and chromosomal aberrations caused by acetaldehyde, a primary product of alcohol [15].

Peake et al. reported that acetaldehyde causes DNA replication stress, leading to the activation of the FA pathway in esophageal keratinocytes [16]. We previously reported that patients with ESCC were enriched in *ALDH2*-associated mutational signature 16, which has a high contribution rate to *ALDH2* mutations related to alcohol metabolism [5].

Considering that germline pathogenic variants in *FANC* genes were found in the two ECS cases in this study, the potential role of the genetic association between the FA pathway, acetaldehyde accumulation, and carcinogenesis in esophageal cells is intriguing.

Our findings suggest that genomic abnormalities in somatic and germline cells contribute to the etiology of ECS in Japanese patients. However, this study was limited due to the small size with only two cases. Genomic analysis studies with a larger sample size should be performed in the future to validate our findings.

## Conclusion

We reported on WGS for genomic analysis of ECS. Our findings indicate that therapeutic drugs targeting the activation signal or the FA pathway might be effective in treating ECS, however, the therapeutic significance should be elucidated in future studies.

### Supplementary Information

Supplementary Material 1.

Supplementary Material 2.

## Data Availability

After de-identification, individual participant data that underline the results will be shared with the investigators when the proposed use of the data is approved by the investigators from the Shizuoka Cancer Center identified for this purpose. Proposals should be directed to y.tsubosa@scchr.jp.

## Abbreviations

CN: Copy number
CT: Computed tomography
ECS: Esophageal carcinosarcoma
ESCC: Esophageal squamous cell carcinoma
FA: Fanconi anemia
GEP: Gene expression profiling
SNV: Single-nucleotide variants
TMB: Tumor mutational burden
WES: Whole-exome sequencing
WGS: Whole-genome sequencing
