# Supplementary material for: Genomic alterations in two patients with esophageal carcinosarcoma identified by whole genome sequencing: a case report
Source: Surg Case Rep. 2024 Aug 19;10:191. doi: 10.1186/s40792-024-01978-8 (PMC11333669; doi:10.1186/s40792-024-01978-8)
Supplement: Supplementary file 2 — Supplementary Material 2. [file 40792_2024_1978_MOESM2_ESM.pptx]

## Slide 1
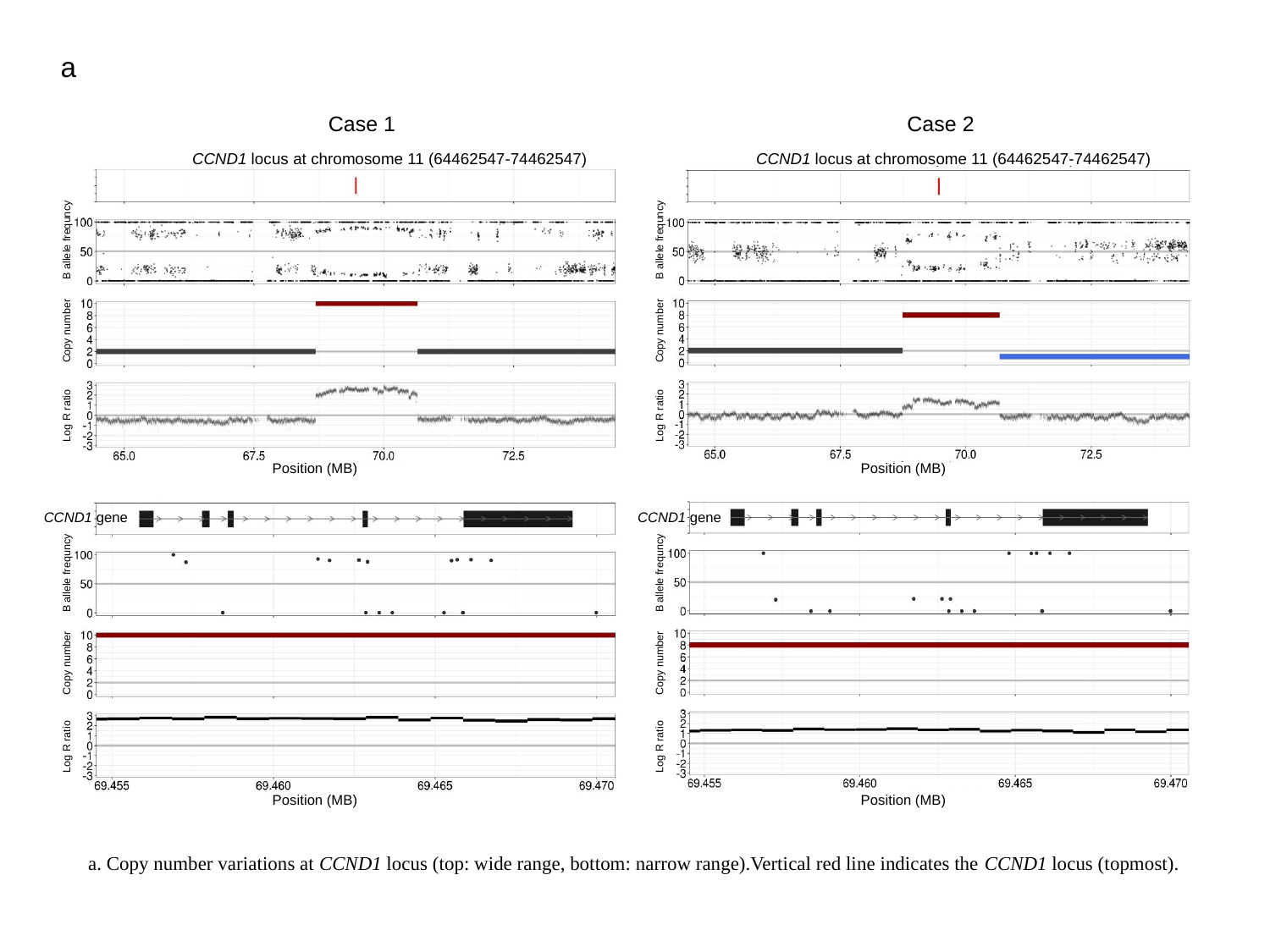

a
Case 1
Case 2
CCND1 locus at chromosome 11 (64462547-74462547)
CCND1 locus at chromosome 11 (64462547-74462547)
B allele frequncy
B allele frequncy
Copy number
Copy number
Log R ratio
Log R ratio
Position (MB)
Position (MB)
CCND1 gene
CCND1 gene
B allele frequncy
B allele frequncy
Copy number
Copy number
Log R ratio
Log R ratio
Position (MB)
Position (MB)
a. Copy number variations at CCND1 locus (top: wide range, bottom: narrow range).Vertical red line indicates the CCND1 locus (topmost).

## Slide 2
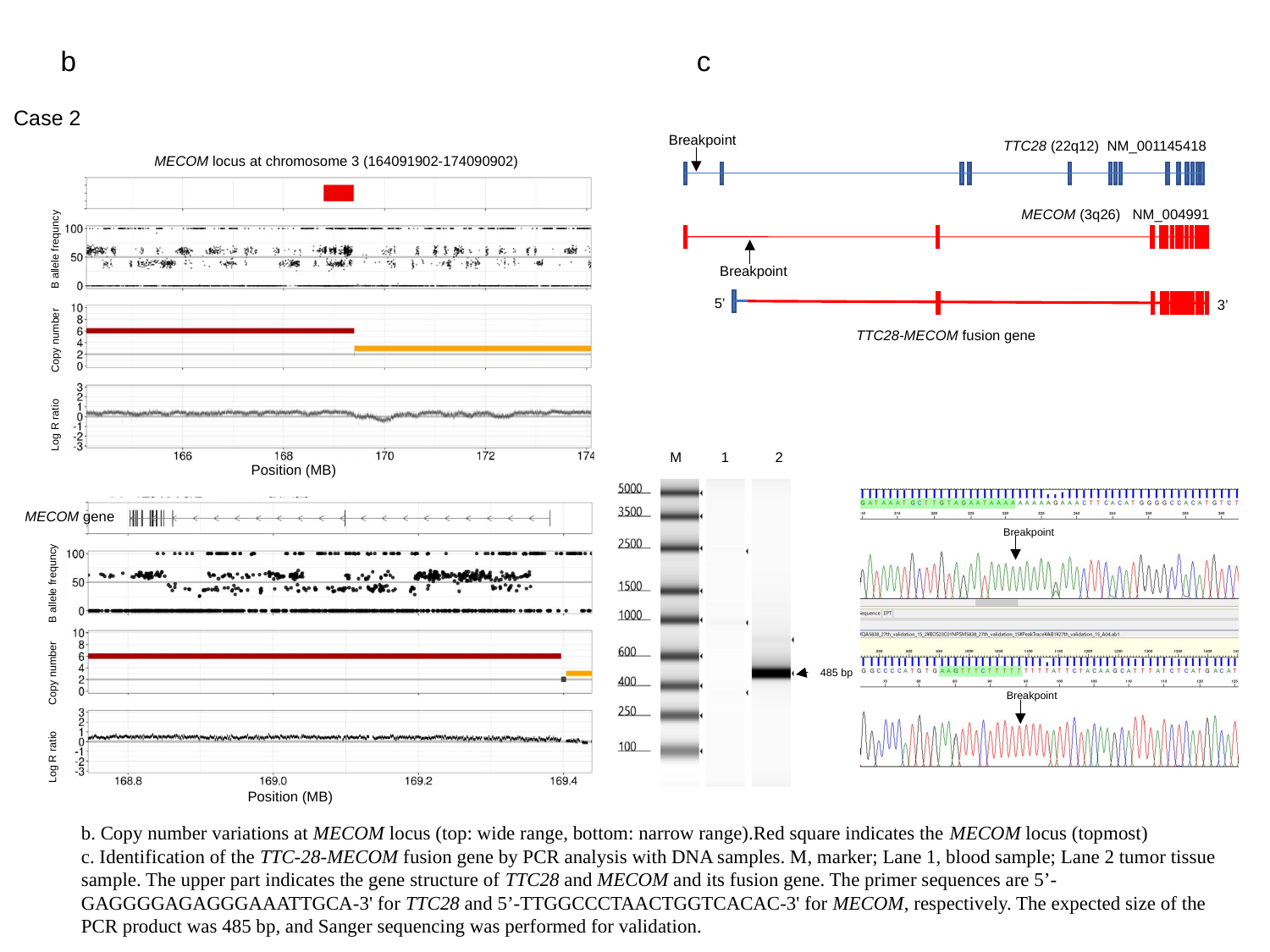

b
c
Case 2
Breakpoint
TTC28 (22q12) NM_001145418
MECOM (3q26) NM_004991
Breakpoint
5’
3’
TTC28-MECOM fusion gene
MECOM locus at chromosome 3 (164091902-174090902)
B allele frequncy
Copy number
Log R ratio
M
1
2
485 bp
Position (MB)
Breakpoint
Breakpoint
MECOM gene
B allele frequncy
Copy number
Log R ratio
Position (MB)
b. Copy number variations at MECOM locus (top: wide range, bottom: narrow range).Red square indicates the MECOM locus (topmost)
c. Identification of the TTC-28-MECOM fusion gene by PCR analysis with DNA samples. M, marker; Lane 1, blood sample; Lane 2 tumor tissue sample. The upper part indicates the gene structure of TTC28 and MECOM and its fusion gene. The primer sequences are 5’-GAGGGGAGAGGGAAATTGCA-3' for TTC28 and 5’-TTGGCCCTAACTGGTCACAC-3' for MECOM, respectively. The expected size of the PCR product was 485 bp, and Sanger sequencing was performed for validation.
